# Multi-century (635-year) spring season precipitation reconstruction from northern Pakistan revealed increasing extremes

**DOI:** 10.1038/s41598-023-50819-5

**Published:** 2024-01-02

**Authors:** Nasrullah Khan, Narayan Prasad Gaire, Oimahmad Rahmonov, Rafi Ullah

**Affiliations:** 1 Department of Botany, University of Malakand, Dir Lower, P.O. Box 18800, Chakdara, Khyber Pakhtunkhwa Pakistan; 2 Department of Environmental Science, Patan Multiple Campus, Tribhuvan University, Lalitpur, Nepal; 3 Faculty of Natural Sciences, Institute of Earth Sciences, University of Silesia in Katowice, Katowice, Poland

**Keywords:** Climate sciences, Ecology, Environmental sciences

## Abstract

The Hindu Kush Himalaya region is experiencing rapid climate change with adverse impacts in multiple sectors. To put recent climatic changes into a long-term context, here we reconstructed the region’s climate history using tree-ring width chronologies of climate-sensitive *Cedrus deodara* and *Pinus gerardiana*. Growth-climate analysis reveals that the species tree-growth is primarily limited by moisture stress during or preceding the growing season, as indicated by a positive relationship between the chronology and precipitation and scPDSI, and a negative one with temperature. We have reconstructed 635 years (1384–2018 CE) of February–June precipitation using a robust climate reconstruction model that explains about 53% variance of the measured precipitation data. Our reconstruction shows several dry and wet episodes over the reconstruction period along with an increase in extreme precipitation events during recent centuries or years. Long, very wet periods were observed during the following years: 1392–1393, 1430–1433, 1456–1461, 1523–1526, 1685–1690, 1715–1719, 1744–1748, 1763–1767, 1803–1806, 1843–1846, 1850–1855, 1874–1876, 1885–1887, 1907–1909, 1921–1925, 1939–1944, and 1990–1992, while long dry periods were observed during the following years: 1398–1399, 1464–1472, 1480–1484, 1645–1649, 1724–1727, 1782–1786, 1810–1814, 1831–1835, 1879–1881, 1912–1918, 1981–1986, 1998–2003, and 2016–2018 CE. We found predominantly short-term periodicity cycles of 2.0, 2.2, 2.3, 2.4, 2.6–2.7, 2.9, 3.3, 4.8, 8.1–8.3, and 9.4–9.6 years in our reconstruction. Spatial correlation analyses reveal that our reconstruction is an effective representation of the precipitation variability in the westerly climate-dominated areas of Pakistan and adjacent regions. In addition to the influence of regional circulation systems like western disturbances, we found possible teleconnections between the precipitation variability in northern Pakistan and broader-scale climate modes or phases like AMO and ENSO. The study also highlights the prospects of tree-ring application to explore linkages between western disturbance, increasing intensity and frequency of extreme climate events, and analysis of long-term atmospheric circulation over the western Himalayan region.

## Introduction

Climate change is a vital issue globally because of its adverse impacts in many sectors^1^. The pace of climate change in the Hindu Kush Himalaya (HKH) is faster than the global average and many other regions of the world^2^. The Hindu Kush Karakorum Himalaya (HKKH) mountains, also known as the Asian freshwater tower, deliver a wide range of ecosystem services and are the basis for the livelihoods of 220-million people living in the region, as well as indirectly of over a billion people living in the downstream river basins^3,4^, which are highly affected by climate change because this region is experiencing a rapid temperature increase, extreme precipitation, and intense and frequent drought and flood events^5–8^. However, studies from the Karakorum region have shown less persistent results such as some glaciers advancing, and a temperature that is increasing/decreasing^9^. The Hindu Kush Karakorum and Western Himalaya (HKKWH) has seasonality in its precipitation distribution, with a major contribution of westerlies (also known as western disturbance) during winter and spring seasons and a minor contribution of Indian summer monsoon during summer and to some extent by orographic precipitation^6,7,10–13^. Studies have shown a decreasing trend of western disturbance days precipitation and but increasing trend of non-western-disturbance days precipitation in the Himalaya region during recent years^14^ Since HKKWH receives majority of the annual precipitation throughout winter and spring months, water availability during this season is crucial for multiple processes, including tree growth. Likewise, the discharge of perennial and ephemeral rivers found in the region is governed by this precipitation seasonality^3,7,15,16^. A sufficient amount of precipitation is not only important for the natural ecosystem to function in the Himalaya region but also for irrigation, hydropower generation, and other domestic day-to-day needs of society. Since climate change is severely affecting the HKKH region, impacts on water, agriculture, and energy resources and overall livelihood of people of the region are foreseen to be aggravated further in the future^17^. Agriculture is one of the main contributors to the national GDP of Pakistan, and long-term information on the water availability in different seasons is crucial to sustaining the economy of the country^18^. Though there are about 300 meteorological stations in Pakistan, most of them are situated at low elevations or on valley bottoms, with short records which are insufficient for long-term trend analysis and for analyzing the periodicities in precipitation distribution^19,20^. Increasing extremes in precipitation have increased the risk of water-induced disasters like floods or droughts because Pakistan and other Himalayan countries have experienced disastrous floods and severe droughts in recent years^5,6,21^. Therefore, knowing the long-term climatic history and dynamics especially precipitation of the region is imperative.

Tree-rings are a valuable and proven source of high-resolution proxy climatic data on a centennial to millennial scale for data deficient regions^22^. Consequently, tree-ring approach have been used extensively for reconstructing centennial to millennial temperature, precipitation, drought indices, and stream flow, among others^7,23–25^. Several tree rings based seasonal to annual precipitation reconstructions have been carried out across the HKKH^24,26–31^, and contiguous regions^32–34^. Among precipitation reconstructions from the Himalayan region, large numbers of them are from the Indian western Himalayas, including the Kashmir region. Pakistan holds several tree species which are promising for long-term hydro-climatic studies^35,36^. Using this fact, a few tree rings based seasonal to annual temperature^7,30,37,38^, precipitation^39^, drought index^38^ and streamflow^25^ reconstructions have been carried out in different areas of northern Pakistan. Temperature reconstructions from the Karakorum region have been mentioned from in-phase^30^ to out-of-phase^38^ trends, to the northern hemispheric temperature trend. Compared to other regions, very limited precipitation reconstructions can be found in Pakistan. Treydte et al.^39^ reconstructed annual (October–September) precipitation by using *Juniper* trees annually resolved oxygen isotope ratio chronologies. Likewise, annual (June–May) precipitation reconstruction (1540–2016 CE) from the Karakoram region in Pakistan divulges an out-of-phase relationship in rainfall between the southern and northern slopes of the Hindu Kush Karakorum Western Himalaya region ^8,31^. These annual precipitation reconstructions do not necessarily reflect the actual trend and pattern in the seasonal precipitation across Pakistan as there is strong seasonality as well as spatial variability in precipitation distribution. Northern Pakistan receives the majority of its total annual precipitation during winter and spring months and seasons. The water received during winter and spring seasons is crucial for multiple areas, including forests, vegetation, tree growth, agriculture, and irrigation, drinking water, and hydropower generation. A large irrigation network exists in northern Pakistan, and millions of livelihoods are directly dependent on the agriculture sector. The Hindu Kush Karakorum Himalaya is experiencing a different pace of climate change within Pakistan^5,29,40^. However, the available climate reconstructions are still not sufficient to represent topographically heterogeneous and climatically diverse regions in Pakistan since station data analysis has revealed spatial differences in the effects of climate change. Therefore, more seasonal climate reconstructions are necessary to provide a robust picture of the long-term climate variability and change, which can provide a scientific basis for the formulation of appropriate policies and programs for water-related sectors in the historical context. Therefore, in this study, we aim to develop a new site and regional composite of tree-ring chronologies from multiple species and to reconstruct the multi-centennial long precipitation history of northern Pakistan.

## Materials and methods

### Study area

**Figure 1:**
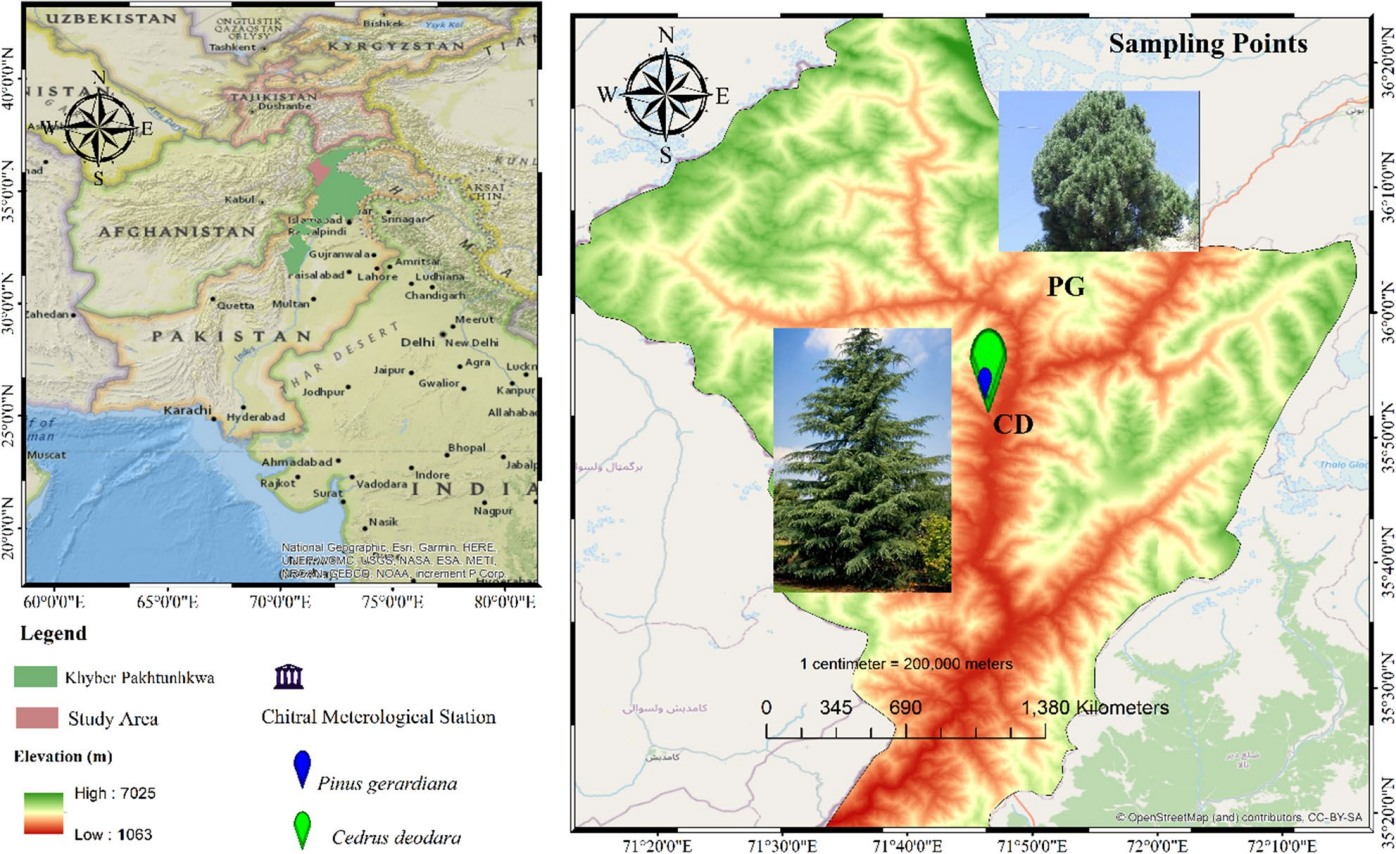
Location of the sampling sites, species sampled and climate station used in this study.

We carried out this study in Chitral which is the largest district in Khyber Pakhtunkhwa Province Pakistan (Fig. 1). The forests growing in the study area are dominated by *C. deodara*, *P. gerardiana*, and *P. wallichiana*, as well as several other broadleaved species.

**Figure 2:**
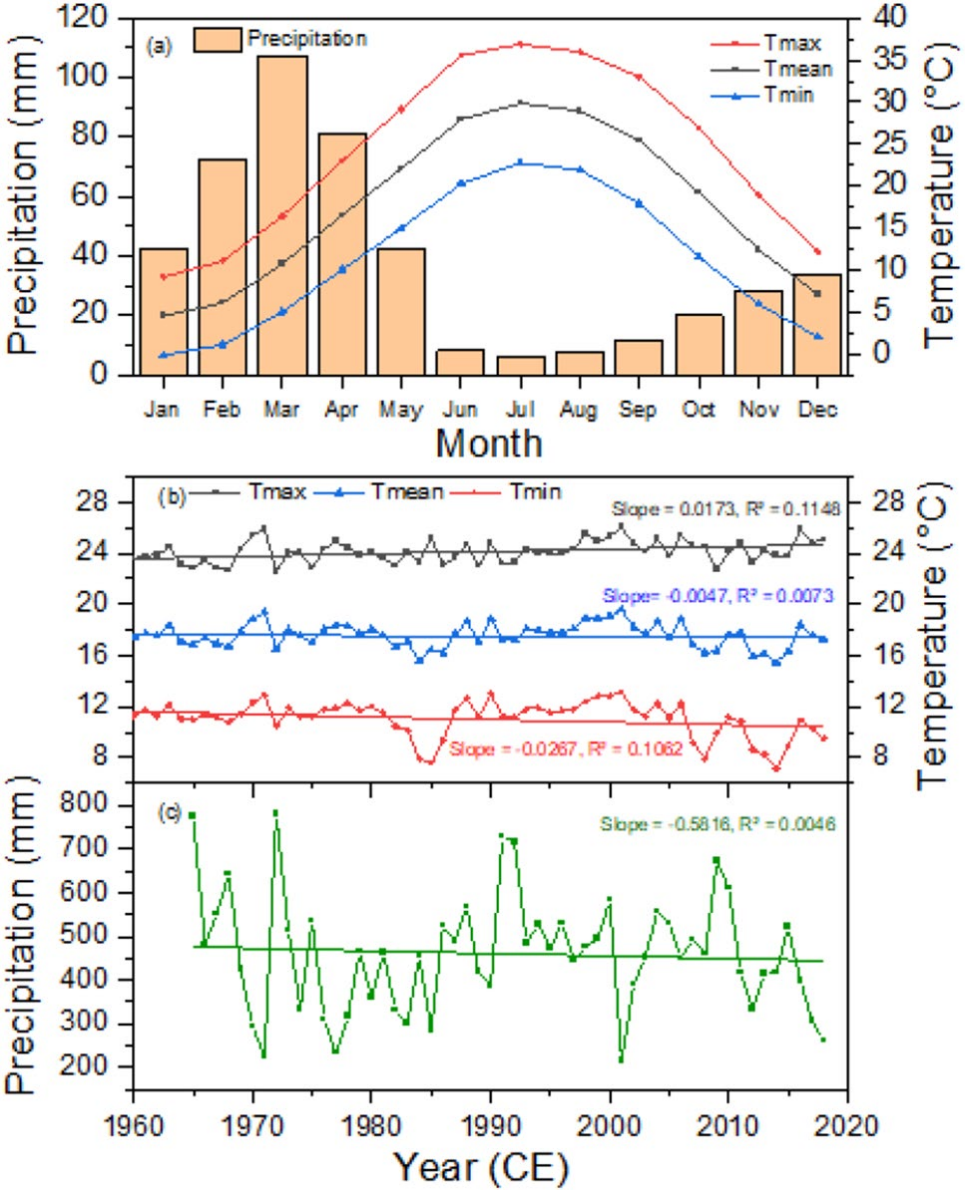
Climograph showing monthly climate at the study area (**a**) with temporal trend in the annual temperature (**b**) and precipitation.

The climate of the region is temperate and is predominantly influenced by a westerly wind with dry summers and wet winter and spring seasons (Fig. 2). The average annual rainfall at Drosh station, close to the present study site, is approximately 460 mm from 1965 to 2018 CE, and the annual mean temperature is 17 °C with a maximum temperature of 36.9 °C. The lowest temperature was − 1.6 °C. The coldest months are December to February, while June–August is largely the hottest months (Fig. 2). The CRU and station climate data have shown no significant trend in the precipitation or the mean and minimum temperatures, but the maximum temperature is increasing significantly (Fig. 2).

### Field visits and sample collection

The field visits were conducted, and tree cores were collected from *C. deodara* and *P. gerardiana* following the commonly used standard dendrochronological sampling procedure ^41,42^. We took the core samples from the south and southwest aspects of the mountain slopes. Mostly, two cores were collected per tree (4.5 feet above ground level) parallel to the contour, while in some trees where the collection of two cores was not feasible due to the steep slope only one sample was collected. In the case of *C. deodara*, cores were collected from both living and dead trees, while *Pinus* samples were collected from living trees only. More than 300 cores of both the types of conifer trees were collected.

### Laboratory analysis of samples and chronology development

Tree cores were analyzed in the lab following standard procedure^41,42^. The collected tree increment cores were air-dried and subsequently glued into grooved wooden frames. The samples were left to dry over a few days. Then, each core was sanded and polished using a sanding machine, followed by hand-polishing using different grades of sandpaper until the rings became clearly visible to study under a stereo microscope. Every ring of core samples was counted under the stereo-zoom microscope and then dated to the exact calendar year of formation based on the known date of the formation of the outer rings based on the sampling date and verified after cross-dating. After dating the tree-ring sequences, we measured the width of each ring to the nearest 0.01 mm accuracy with the LINTAB5™ measuring system attached to a computer with a TSAP-win program^43^. All tree cores were cross dated by matching patterns of relatively wide and narrow rings to account for the possibility of ring-growth anomalies such as missing rings, false rings, or measurement errors^41^. Each tree-ring width series was visually (using graphs) and statistically (using Gleichläufigkeit, t-values, and the cross-date index-CDI) cross-dated using the software package TSAP–Win^43^. The accuracy of cross-dating and other measurements was further checked using the COFECHA program and necessary correction was done^43^. Raw tree-ring data contains climatic and biological growth signals on it^41^. Detrending of cross-dated raw series was done to remove the trend related to the tree age and geometry and to maximize the common signal likely related to climate. The standardization was done by fitting a modified negative exponential growth curve in each raw series using the computer program dplR^44^. The residual chronology was computed by removing the autocorrelation effect. We then computed the final chronology using a robust bi-weight mean^37^. Various chronology statistics were calculated to evaluate the quality and dendroclimatic suitability of the developed individual site chronologies and regional composite chronologies^42,45^. The running R-bar and EPS value was calculated in the RCSsigfree program (https://www.ldeo.columbia.edu/tree-ring-laboratory/resources/software).

### Growth-climate response analysis

Available climatic data (both stations and CRU grid) from the areas covered by each tree-ring chronology were collected and used for growth climate response analysis^46–49^. To establish the relationship between growth and climate, simple and bootstrapped correlation analyses were carried out between climate (temperature and precipitation) and drought index (scPDSI) and each tree-ring site chronology for the period of overlap with climatic data (1960–2016) using treeclim R package ^42^. 1000 bootstrap samples are taken from the original distributions of climate and tree-ring data, either using the stationary bootstrap or classical bootstrap^42^. A 17 months dendroclimatic window starting from previous year June to current year October along with different seasonal combinations of the monthly data was used to identify growth-limiting climatic factors. Furthermore, we analysed the seasonal climate effect on radial growth by doing 'seascorr' analysis, using Pearson’s partial correlation between the chronology and climate data, taking precipitation as the main variable and temperature as the secondary variable and repeating the analysis using vice-versa climate variable. The 'seascorr' analysis was done using the 'treeclim' package in R^50^. The moving correlation between the chronology and climate data was also analyzed in the 'treeclim' package^50^ to check the temporal stability of the growth-climate response and to check possible divergence issues. 'seascorr' analysis (Seasonal partial correlation) calculate seasonal correlation with primary and secondary climate variables and tree-ring data^50^. Empirical non-exceedence probabilities are used to test the coefficients of the response/correlation function with the original data for significance^50^.

### Regional composite chronology development

To check the common climatic signal captured in the individual site chronologies, the Pearson correlation between each site chronology was analysed for the full common period (1400–2016) and recent period (1900–2016). Similarly, the principal component analysis (PCA) of these three site chronologies was also computed using the ‘FactoMineR’ by Lê et al.^51^ to see the common signal captured on them After checking the correlation value, variance explained in first principal component (PC#1), and climatic response of the individual site chronologies with station and CRU data, it was found that they can be combined for regional composite chronology. Therefore, all raw tree-ring data of each site chronology were combined in a single file and used to develop the composite chronology. The same detrending method which was used in the development of individual site chronology was applied to the development of composite chronology. Then, the climatic response of the composite chronology was analysed. Similarly, the climatic response of the PC#1 value chronology was analyzed to check common climatic signals. Since the composite chronology showed a stronger response than the PC#1 chronology, at the end, the composite chronology was used for climate reconstruction purposes.

### Reconstruction method of regional precipitation

From the growth-climate response analysis, the primary growth limiting climatic factor was identified. Then, the significant response was used to develop a growth-climate model which was subsequently used to reconstruct the past climate of target variable and target season. From the growth-climate relationship analysis between the regional composite chronology and climatic data, it was found that spring season (February–June, FJ) precipitation reconstruction is theoretically possible; so, it was targeted for reconstruction. The transfer function explained by Blasing and Fritts ^33^ was used to reconstruct the climatic variable (Precipitation). Finally, one reconstruction model was built using simple linear regression using spring season (February–June) precipitation (1965–2018 CE) as the predictant (dependent variable), and the regional composite tree-ring chronology as the independent variable (predictor). The time-stability of the model was tested using calibration and verification methods i.e., split-sample-half validation^52^ and leave-one-out cross-validation^53^. Pearson’s correlation coefficient (r), the coefficient of determination (R^2^), adjusted coefficient of determination (R^2^_adj_), reduction of error (RE), and coefficient of efficiency (CE) were used to evaluate model skill. We also calculated root-mean squared-error (RMSE) from the model as an indicator of variability in the reconstruction. Linear regression model assumptions were evaluated by inspection of residual plots to ensure that there was no pattern in error variance. The autocorrelation function of the residuals was examined visually, and the Durbin–Watson statistic was used to evaluate the assumption of independence in the predictor variable. We also conducted a sign test to evaluate the fidelity of year-to-year changes in the reconstructed precipitation to the tree-ring predictor^41^. The RE and CE positive values of climate-growth models was taken as a basis for the validity and reliability of the regression model^37^. Once the model was judged to be effective and stable, it was then applied to reconstruct past spring season precipitation for the period covered by regional composite tree-ring width chronology. The reconstruction was truncated at the point at which the EPS value became less than the arbitrary but commonly used threshold value of 0.85^45^. Spatial representation of the reconstruction was checked in the KNMI climate explorer^33,54^ by performing spatial correlation between the observed and reconstructed precipitation with CRU grid precipitation and sea surface temperature. To check the teleconnection and identify the influence of broader scale climatic modes and phases, relationship (Pearson’s correlation) between the reconstructed climate and different climatic indices (ENSO, AMO, SOI, ONI, NAO, PDO, etc.) was analyzed (Supplementary Table S3). Similarly, we also compared climatic trends in our reconstructed precipitation from Pakistan with other independent and available reconstructions reported from the Himalayas to check the coherencies in the reconstructions. Furthermore, a power spectrum analysis was performed by using the Multi Taper method to check the periodicities in spring precipitation^55^. Similarly, Morlet Wavelet analysis was also performed to check the temporal pattern in the periodicity of the precipitation^56^.

### Experimental research and field studies on plants

a. The use of plant parts in the study is in accordance with international, and national guidelines. b. The collection of plant material and the performance of experimental research on such plants complied with the national guidelines of the Republic of Pakistan.

## Results

### Tree-ring chronologies and their characteristics

**Figure 3:**
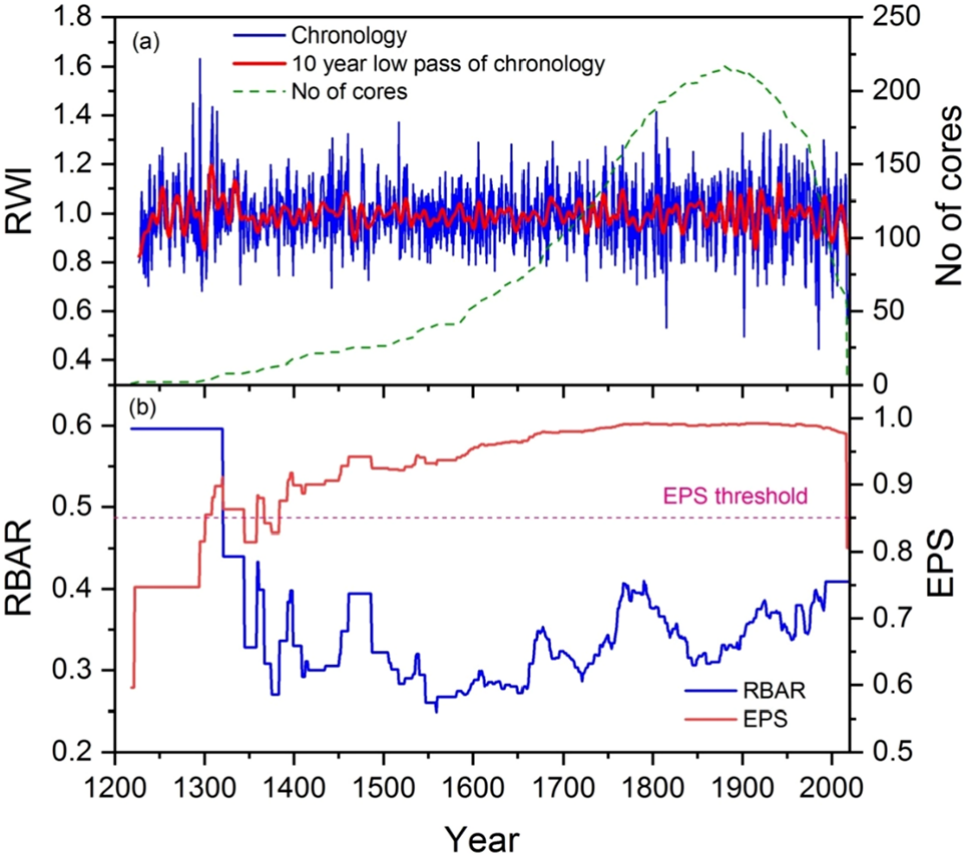
Regional composite tree-ring width residual chronology (blue) from Northern Pakistan along with their 10-year smoothing spline (thick red line) and no of cores (blue dotted line) (**a**). The running EPS (red line) with commonly used threshold value of 0.85 (pink dotted horizontal line) and running R-bar (blue line) are presented in the lower panel (**b**).

Tree-ring width site chronologies were developed using *Pinus gerardiana* and *Cedrus deodara*. The *P. gerardiana* chronology spans from 1218 to 2017 CE, while *C. deodara* chronologies span from 1375 to 2017 CE and 1394–2018 CE (Fig. S1). The commonly used chronology statistics (high R-bar, SD, and a high EPS) indicated that they have captured some common climatic signal and have the dendroclimatological potential of these site chronologies (Table S1). These chronologies have revealed short to medium-term (annual to decadal) fluctuations in growth over time, along with decreasing trends in recent years (Fig. S1). These chronologies capture common climatic signals as revealed by principal component analysis (PCA) because first Principal Component (PC#1) explains 73.72% variance in the data (Fig. S2; Table S2). Similarly, we found a strong correlation between the site chronologies for the common period of 1400 to 2017 CE (r = 0.57–62) and the recent period of 1900–2017 (r = 0.77–0.87). Therefore, a regional composite chronology which spans 1218–2018 CE with high dendroclimatic potential as indicated by high EPS (0.977) and high R-bar (Fig. 3) was developed by combining site chronologies. The composite chronology has crossed the arbitrary but commonly used EPS threshold value of 0.85 since 1384 CE.

### Climatic response of site and composite chronology

**Figure 4:**
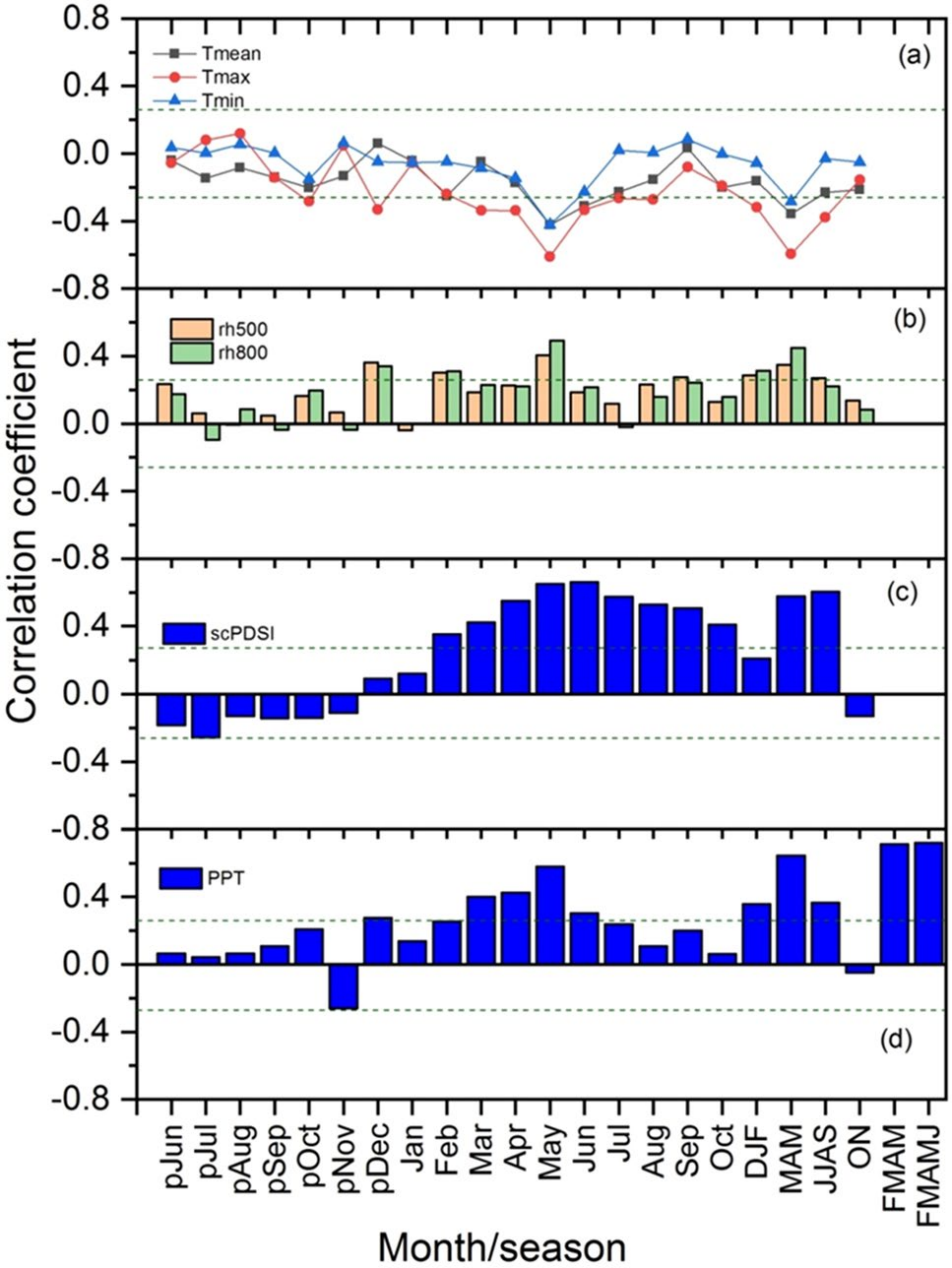
Relationship (correlation coefficient) between the tree-ring composite residual chronology and monthly and seasonal climatic data. The horizontal dashed line represents the significance of correlation coefficients at 95% level.

The annual growth of the studied conifers tree species in northern Pakistan was primarily limited by the availability of moisture during the growing season and months as revealed by growth-climate response analysis (Fig. S3). On the basis of seasonal response, high precipitation availability during all winter, spring, and summer seasons positively favors growth. We found a strong positive correlation between site chronology (standard and residual) and station and CRU gridded precipitation during the spring months and seasons and a negative correlation with the temperature of the same months and seasons (Fig. S3). There was a strong positive correlation between site chronologies and scPDSI during most of the months and seasons, and a strong relationship with spring and summer. The response of standard and residual chronology is almost similar in direction, but the magnitude of response of residual chronology is stronger than standard chronology in most cases (Fig. S3). The growth-climate relationship of regional composite tree-ring width chronology is even stronger than that with individual chronologies (Fig. 4).

**Figure 5:**
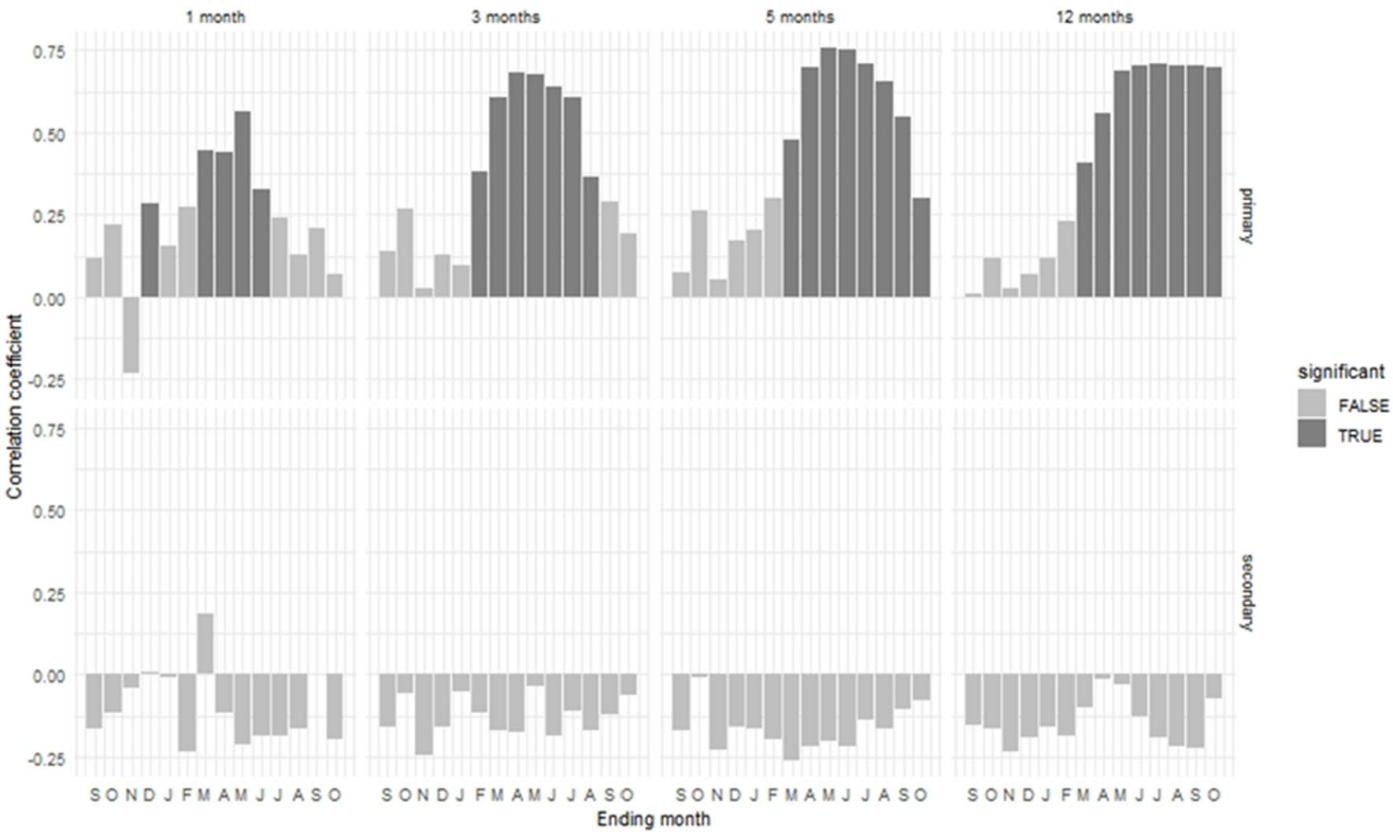
Seasonal correlation (seascorr) between the composite chronology and Drosh station precipitation and temperature data. Precipitation is primary and temperature is secondary variable. The analysis is done taking one, three, five, and twelve-month periods. The shaded bar represents significant correlation.

We found a positive relationship between the composite tree-ring residual chronology and precipitation during the winter, spring, and summer seasons, with relative humidity significantly positive during winter and spring, with scPDSI significantly positive during the spring and summer months and seasons (Fig. 4). But there was a negative correlation between the composite chronology and temperature in most of the months and seasons, with a strong negative correlation during the spring and early summer seasons (Fig. 4). The negative relationship with the maximum temperature is stronger compared to the mean and minimum temperatures. Seascorr analysis was carried out using the partial correlation to check the seasonal climate effect on tree-growth (Fig. 5). We found a positive relationship between the chronology and precipitation for most of the time on a monthly, seasonal, and annual basis, which was strong in the spring months and spring summer seasons.

**Figure 6:**
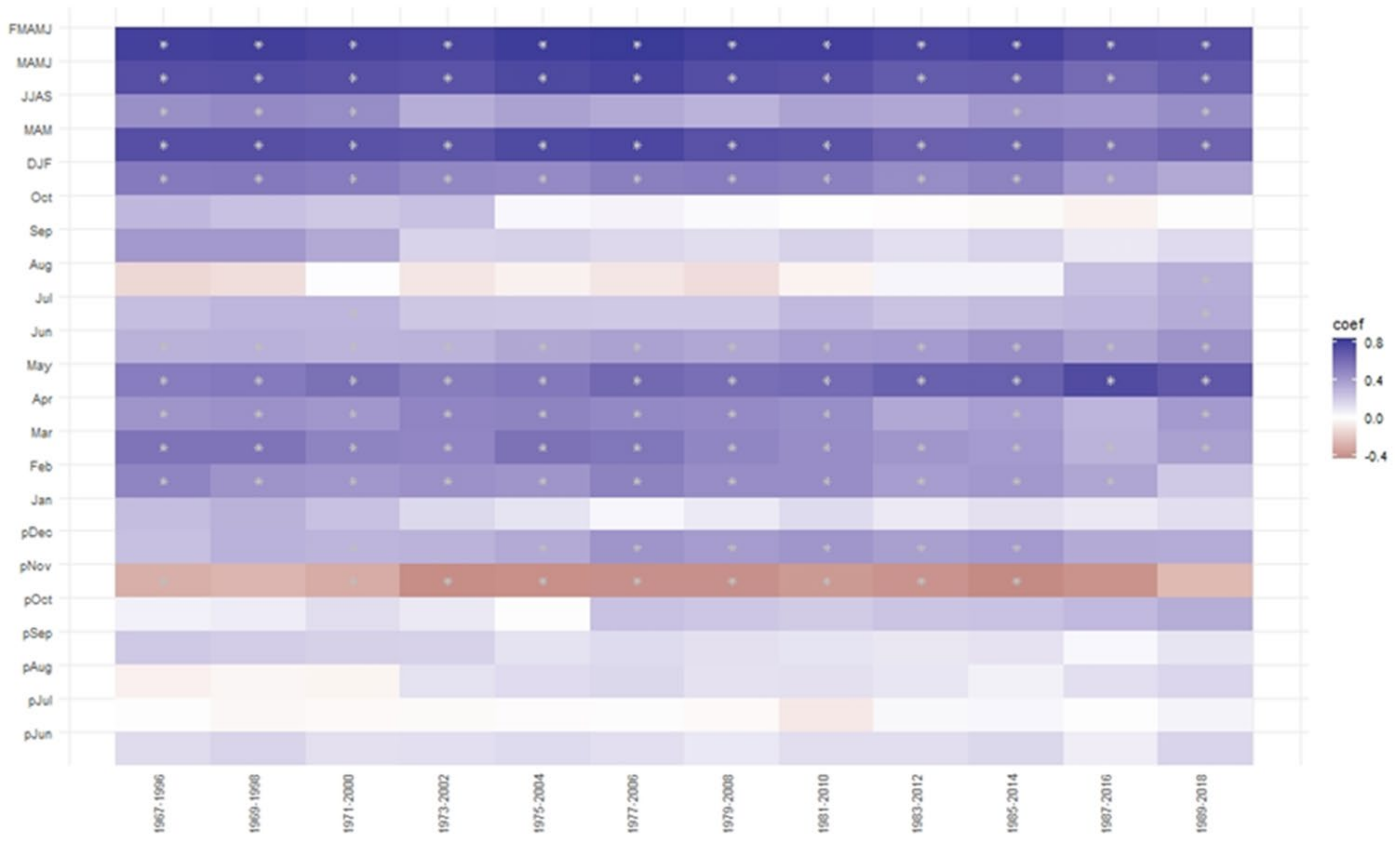
Heat map showing moving correlation between the regional composite tree-ring width residual chronology and monthly and seasonal precipitation of Drosh station from1965 to 2016. The DJF, MAM/MAMJ/FMAMJ, and JJAS represent winter, spring, and summer seasons, respectively. The * symbol in the map indicates significant correlation (analysis was done using the 'treeclim' package in R^50^).

This analysis further confirmed that the growth of *C. deodara* and *P. gerardiana* trees in northern Pakistan is mainly limited by availability of moisture during the spring and summer months and seasons, with a positive relationship with precipitation and a negative one with the temperature of the same months and seasons. The temporal stability of the climatic response of the growth was checked by taking a 30-year climatic moving window with a 2-year window overlap (Fig. S4). The results showed that precipitation and temperature relationships in most of the months were stable throughout the study period (Fig. S4). The relationship between tree-growth and precipitation during the winter and spring seasons was persistently positive and strong (Fig. S4, Fig. 6). This indicates that our growth climate relationship can provide a solid basis for the purpose of climate reconstruction.

### Regional precipitation reconstruction from northern Pakistan

**Figure 7:**
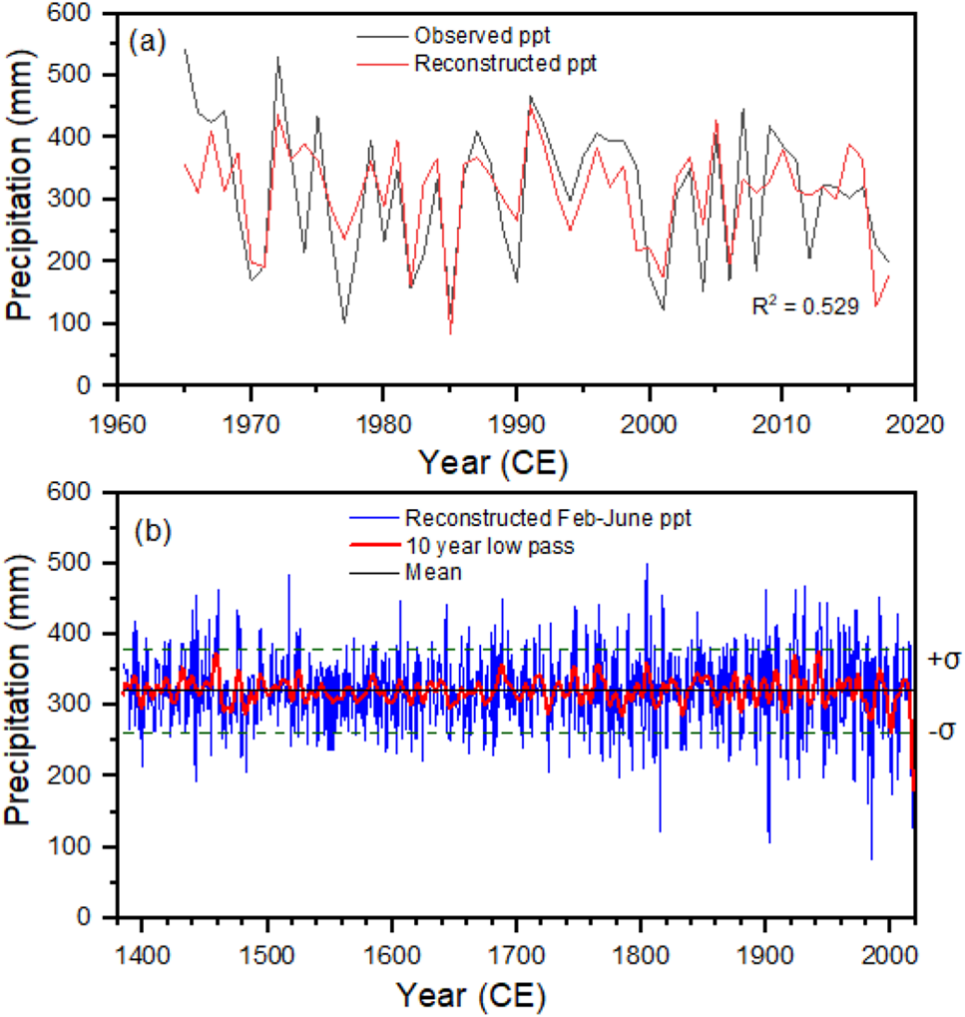
Comparisons between the observed (black line) and reconstructed (red line) spring season (Feb–June) precipitation from Northern Pakistan during the calibration period of 1965–2016. Spring season (Feb–June) precipitation reconstruction from Northern Pakistan (green line) with 10-year low passes (red line). The horizontal dashed lines indicate the precipitation beyond the one standard deviation (σ) from the long-term mean (black horizontal line).

From the growth-climate response analysis, it was found that spring season (February–June) precipitation is the best candidate for reconstruction. Therefore, we developed a reconstruction model using regional composite residual chronology and spring season precipitation and checked the robustness of the model by using LOOCV and split-half validation statistics. Our model is robust as it passed commonly used calibration (R, R^2^, F-stats, p-value, DW) and verification (RE, CE, sign test, product mean test, RMSE) statistics and tests (Table 1, Fig. 7a).

**Table 1:** Leave-one-out cross-validation and split-sample half-validation statistics of the precipitation reconstruction models from western Nepal.

Reconstruction model: Precipitation_Feb–June_ = 430.6*RC − 107.51									
Leave-one-out cross validation statistics									
R	R^2^	R^2^_adj_	RMSE	F-stats	p-value	DW	Sign test (±)	PMT	RE
0.727	0.529	0.520	76.945	58.331	0.000	1.82	43/11	4.087	0.497

Split-sample half validation statistics									
	Calibration (1965–1991)	Verification (1992–2018)	Calibration (1992–2018)	Verification (1965–1991)	Calibration (1965–2018)				
R^2^	0.565		0.496		0.529				
R^2^adj	0.547		0.476		0.520				
R^2^-Verification		0.496		0.565					
RE		0.434		0.531					
CE		0.434		0.531					
PRESS					0.497				

*RC* regional composite chronology, *R* regression coefficient, *R2* coefficient of determination, *SE* standard error, *DW* Durbin Watson statistics, *PMT* product mean test, *RE* reduction of error, *CE* coefficient of efficiency, *PRESS* Predicted Error Sum–of–Square.

The model revealed a 52.9% variance in the actual data during the calibration period (1965–2018 CE). By using this robust reconstruction model (Table 1), a 635-year long (1384–2018 CE) February-June (FMAMJ) precipitation was reconstructed for northern Pakistan (Fig. 7b). The over six century-long February–June regional precipitation reconstruction revealed several distinct wet and dry periods.

In the reconstructed precipitation, 38 extremely wet (mean + 1.5SD) and 38 extremely dry (mean-1.5SD) years were recorded. There were periodic fluctuations in the precipitation with several consecutive extremely dry and wet years. The ten wettest years were 1991, 1931, 1924, 1900, 1816, 1804, 1688, 1517, 1460, and 1443, while the ten driest years were 1985, 1902, 1815, 2017, 1982, 2001, 2018, 1442, 1971, and 1917.

The low-frequency analysis (10-year smoothing spline) revealed several persistent dry and wet periods over the reconstruction period, with precipitation consistently declining during recent decades (Fig. 7b). A long wet period (mean + 1SD of 10-year low pass of reconstruction) was observed during 1392–1393, 1430–1433, 1456–1461, 1523–1526, 1685–1690, 1715–1719, 1744–1748, 1763–1767, 1803–1806, 1843–1846, 1850–1855, 1874–1876, 1885–1887, 1907–1909, 1921–1925, 1939–1944, and 1990–1992, while a long dry period (mean-1SD of 10-year low pass of reconstruction) was observed during 1398–1399, 1464–1472, 1480–1484, 1645–1649, 1724–1727, 1782–1786, 1810–1814, 1831–1835, 1879–1881, 1912–1918, 1981–1986, 1998–2003, and 2016–2018.

**Figure 8:**
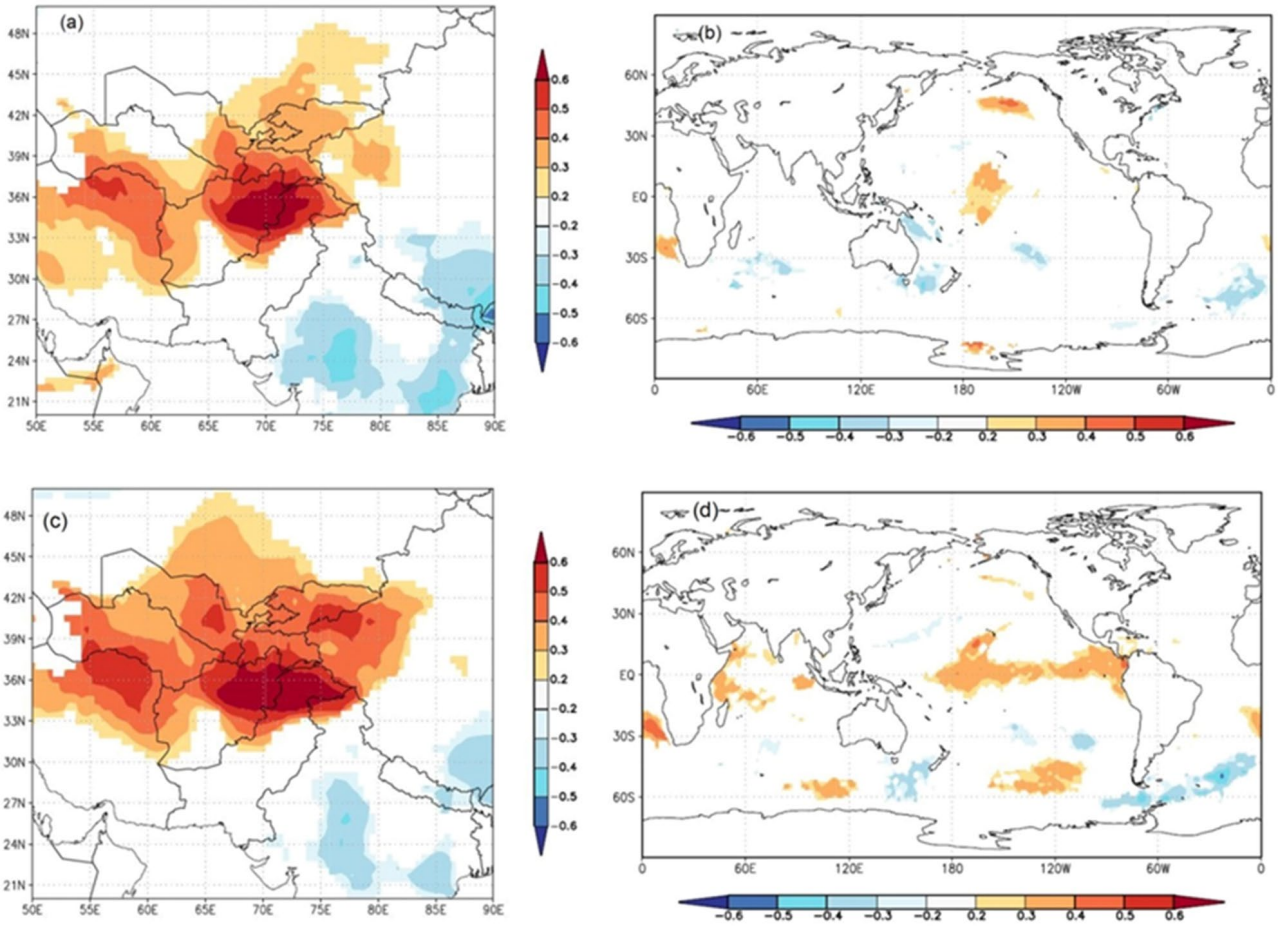
Spatial correlation between observed (**a**) and reconstructed (**b**) spring season precipitation with the CRU gridded precipitation and sea surface temperature (**c**, **d**) for the common period to observed data (1965–2018 CE). The map was prepared online using KNMI climate explorer (https://climexp.knmi.nl/).

The counting of the extreme events in every hundred-year period indicated that the percentage of extremely dry and wet events has been increasing continuously (Fig. S5). Spatial correlation analysis revealed that our precipitation represents the precipitation variability in westerly influenced regions of Pakistan, Afghanistan, Iran, Tajikistan, Turkmenistan, and Uzbekistan, as well as parts of India and of the Tibetan Plateau (Fig. 8).

**Figure 9:**
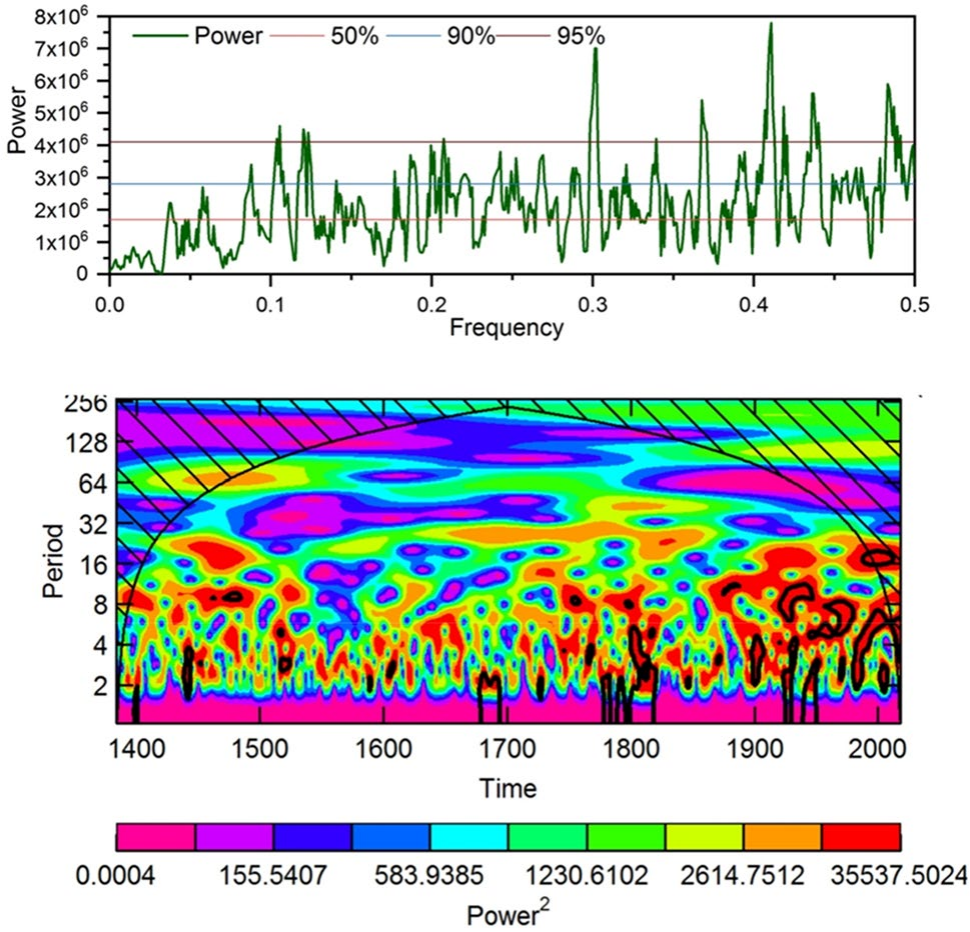
Power spectral (**a**) and Morlet wavelet (**b**) analysis of the reconstructed spring precipitation data from western Nepal Himalaya.

We found a positive spatial correlation between the observed and reconstructed precipitation and CRU grid land precipitation of those regions. Similarly, we found a positive correlation between the observed and reconstructed precipitation from northern Pakistan and the sea surface temperature of the tropical region of the Pacific and Indian Oceans. In addition to the spatial correlation, we analyzed the relationship between (Pearson’s correlation) reconstructed FJ precipitation with different climate indices and modes (Table S3). We found significant positive correlation between the reconstructed precipitation and NINO3.4 and ONI during December to April month. There was a significant negative correlation between the reconstructed FJ precipitation and AMO during May–June Months. Relationship with MEI, PDO and NAO was weak and statistically insignificant in most of the months (Table S3). The power spectral analysis of the reconstructed precipitation data using the Multi Taper method revealed that the Feb–June precipitation in northern Pakistan has mostly short (interannual to decadal) frequency cycles (Fig. 9a). In the reconstructions, significance peaks were found at 2.0, 2.2, 2.3, 2.4, 2.6–2.7, 2.9, 3.3, 4.8, 8.1–8.3, and 9.4–9.6 years (Fig. 9a). Morlet wavelet analysis revealed that the intensity of high frequency cycles is increasing in recent centuries and decades compared to the earlier period (Fig. 9b).

**Figure 10:**
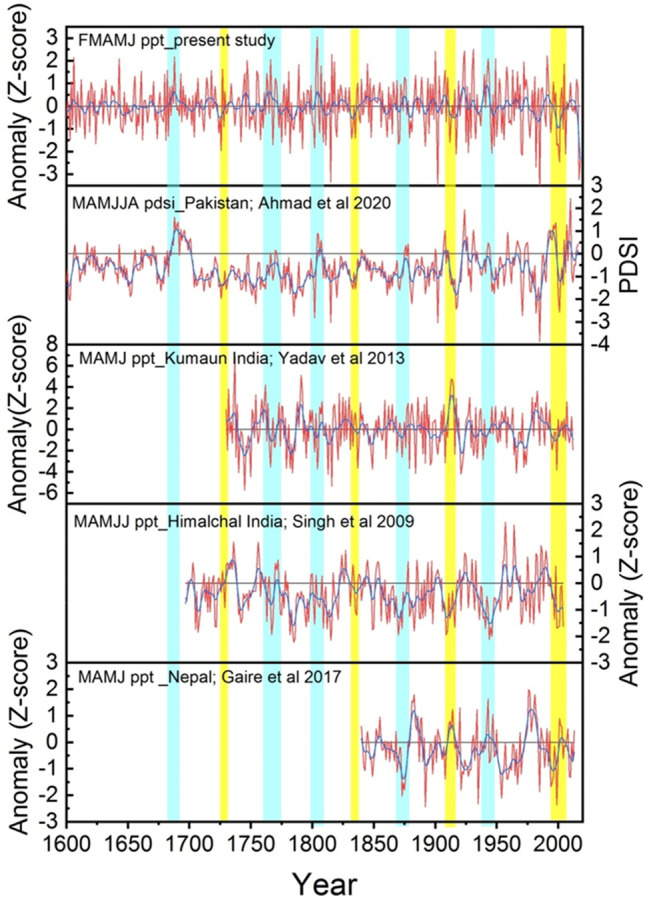
Comparison between February-June reconstructed precipitation (standardized value) from northern Pakistan in our study and other independent reconstructions from the Hindu Kush-Karakorum Himalaya (HKKH) and adjacent region. The compared other reconstructions include March–August PDSI from northern Pakistan^21^, March–June precipitation from Kumaun region in western India Himalaya^86^, March–July precipitation from Himachal region in western India Himalaya^24^ and March-June precipitation from western Nepal Himalaya^30^.

While comparing our present reconstruction with the other independent precipitation and drought index reconstructions from the HKKH and adjacent regions, we found coherencies in the major dry and wet periods, though some site-specific dry and wet periods also exist in different reconstructions (Fig. 10). The most coherent features among the reconstructions are that the spring precipitation has decreased across the HKKH region during recent years and dry episodes around the Tambora eruption period. Cool and warm episodes in reconstructions from the westerly-influenced region are more coherent compared to both westerly and Indian summer monsoon influenced regions (Fig. 10).

## Discussion

### Climatic sensitivities of the tree growth from northern Pakistan

Hindu Kush Karakorum Pakistan Himalaya holds several promising tree species for multi-aspect tree-ring research, including long-term climate reconstruction in the data deficient regions. We found a high dendrochronological potential of the *C. deodara* and *P. gerardiana* tree-ring chronologies with high mean sensitivity, SD, moderate R-bar, and high EPS^41,57^. The chronology statistics we obtained in our study are similar to or even better than those reported for conifer species in previous studies from Pakistan^38,58^ and adjacent regions in the Himalaya^7^. The *C. deodara* and *P. gerardiana* chronologies which we developed in this study are some of the longest chronologies for the respective species from Pakistan. These chronologies follow the climatic pattern with high growth in favorable climates and low growth in unfavorable climates.

The climate-growth relationship analysis revealed that *P. gerardiana* and *C. deodara* trees growing in the northern Pakistan region were mainly limited due to the moisture availability during the growing and proceeding to the growing seasons. We found a positive relationship between the site and composite chronologies and precipitation during most of the months and seasons, with strong relationships with the winter and spring months, and a negative relationship with temperature in the same months and season. This indicates that moisture stress limits tree growth, a fact which was further supported by the strong positive relationship between the growth and drought indices (scPDSI) during the spring and summer months. Seascorr analysis between the chronology and precipitation and temperature data further confirmed that moisture stress (either due to low precipitation or induced by high temperature) limited tree growth. The climate of the northern Pakistan is primarily influenced by westerly winds during winter and spring^10,11,14^ and present study area receives about 80% of its annual precipitation during the winter and spring months (Fig. 2a), which is the ultimate source of moisture for plant growth. Regions of Pakistan at higher elevations receive precipitation in the form of snow and melting, which recharges the groundwater and also water flow. This precipitation provides a moisture supply for tree growth during the spring and early summer months. During the late spring or early summer months, the temperature in the region increases very rapidly, which increases water demand for transpiration^38,41,57^ Studies indicated weakening influence of western disturbance days precipitation in winter and spring season in the HKKH region during recent years^14^. If the area receives less precipitation during the winter and spring months and seasons, there is a water shortage for tree-growth during the growing season, which can lead to the formation of narrow rings during those years^38,41^. The positive relationship with the precipitation and drought index and the negative one with temperature are similar to the observations in some previous tree growth climate studies from Pakistan^58^. Spring or spring–summer season moisture stress in tree growth is widely observed across the Himalayas, particularly in the western Himalaya in India and central Himalaya in Nepal in multiple species^6,7,28–31,59^. Topography of the Pakistan is very diverse and heterogamous with presence of diverse landforms influenced by different climate domains. Therefore, response of tree-growth to temperature is not uniform across the whole of Pakistan because some trees from high elevation regions are showing positive responses to temperature, while those from dry sites are showing negative responses to temperature. Similar to the present study, a negative response of *Picea smithiana* with the spring season temperature has been reported in the Karakoram region of northern Pakistan^60^, with the same for the *C. deodara* from the Hindu Kush range of Pakistan^61^. Similarly, Zafar et al.^62^ found a negative relationship between temperatures and the *P. smithiana* and *P. gerardiana* chronologies from the Gilgit and Hunza valleys of northern Karakorum during most of the months of recent years. But a positive relationship between temperature and *Abies pindrow*, *P. smithiana*, and *P. wallichiana* growth has been found in some sites in northern Pakistan^58^, as well as with *P. wallichiana* trees in the Bagrot valley^63^. Overall, tree growth limited by moisture stress is widely observed in Pakistan and adjacent regions.

### Characteristics of multi-centennial precipitation reconstructions

Tree-rings act as very important proxy climatic sources in various regions of the world^7,38,41,64,65^. We developed a climate reconstruction model using climate and tree-ring data from Pakistan. Our precipitation reconstruction model is robust and reveals a 53% variance in the actual data for the calibration period (1965–2018), which is a comparable or even higher variance explained than the previous tree ring-based climate reconstructions from Pakistan^9,61,64–66^, The temporally stable growth-climate responses, especially those with precipitation, indicate that there is no divergence issue in the growth-climate response, which also supports the robustness of our reconstruction model and reliability of our reconstruction. Hence, we reconstructed a 635-year-long February-June precipitation record from northern Pakistan.

Our more than six-century long (1384–2018 CE) February–June regional precipitation reconstruction revealed several wet and dry episodes (explained in terms of beyond 1 standard deviations (SD) from the long-term mean) in northern Pakistan. Since our reconstruction is based on residual chronology which mainly preserves high frequency signals, we mainly found relatively short increasing and decreasing phases in precipitation. The precipitation has rapidly decreased during recent decades, along with increasing extremes. The extremely wet and dry years found in the present study have also been reported in the station-based studies from Pakistan^5,67^. The recent decreasing trend in precipitation has been a shared observation among several studies in the Himalayan region^28,30,59^. Though precipitation has some local distribution patterns, some of the dry and wet episodes in our study match the dry and wet periods in Pakistan and adjacent HKKH regions^9,28,30,59^. In a 477-year annual (June–May) precipitation reconstruction from Pakistan, Norris et al.^9^ found dry episodes (rainfall below the average) occurred from 1569 to 1577, 1598–1612, 1621–1621, 1638–1654, 1673–1680, 1697–1720, 1728–1739, 1753–1761, 1777–1793, 1801–1840, 1860–1874, 191–1932, 1960–1985, and 1998–2011, while wet episodes (above average) occurred from 1540 to 1568, 1578–1597, 1613–1620, 1632 t–1637, 1655–1672, 1681–1696, 1721–1727, 1740–1752, 1762–1776, 1794–1800, 1841–1859, 1875–1913, 1933–1959, and 1986–1997. Though not all dry and wet episodes perfectly match with our reconstructions due to differences in the seasons and reconstruction locations and the delineation of dry and wet episodes, most of the dry and wet episodes are shared in both studies. Ahmad et al. ^57^ recorded nine dry periods (1593–1598, 1602–1608, 1631–1645, 1647–1660, 1756–1765, 1785–1800, 1870–1878, 1917–1923, and 1981–1995), and eight wet periods (1663–1675, 1687–1708, 1771–1773, 1806–1814, 1844–1852, 1932–1935, 1965–1969, and 1990–1999) in their 424-year March–August PDSI reconstruction from northern Pakistan. We found most of these dry and wet episodes match our reconstruction. Some dry periods found in the present study were also found in the precipitation reconstructions from the Nepal Himalaya^30^. Moreover, we compared our reconstructions with the hydro-climate reconstruction from western Himalaya in India^68–71^ which revealed some coherencies in the dry and wet period (Graph not shown). We found significant positive relationship (correlation) between the present precipitation with that reconstructed from the Indian Himalaya^70,71^ indicating similar climatic driver affecting the western Himalaya. Though we did not find full dry episodes during any of the four historical drought periods across monsoon Asia region (the Ming Dynasty drought: 1638–1641, the Strange Parallels drought: 1756–1768, the East India drought: 1790–1796, and the late Victorian Great Drought: 1876–1878), which have been observed in the Monsoon Asia Drought Atlas^72^, we observed below average to some extremely dry years during those periods. The mismatch between some of the dry and wet episodes in our reconstructions with that in precipitation in western India and Nepal Himalaya could be due to the differential influence of westerly and Indian summer monsoons in those regions. Some discrepancies with MADA could be due to the differential influence of westerly and Asian summer monsoons in our study region and MADA-focused region. Spatial correlation analysis further revealed that our reconstruction effectively captures the precipitation variability in westerly-influenced northern Pakistan, Afghanistan, Turkmanistan, Uzbekistan, Iran, western Indian Himalaya, and parts of the Tibetan Plateau. We found similar (in‐phase) pattern in rainfall in Hindukush‐Karakorum‐Western Himalaya region and adjacent westerly influenced regions in Asia.

Climate and their anomaly of the HKKH and adjacent regions is largely governed by prevailing local to regional general circulation systems in addition to the influence of climatic modes and phases like AMO, ENSO, PDO, and SOI of remote location^10,11,13,14,21,28,30^. However, preservation of the climatic signal, periodicity or spectral peak in tree-ring based climate reconstructions largely depend on method of detrending the raw tree ring data and type of chronology used for the reconstruction^37,73^. The power spectral and wavelet analysis revealed that the over six-century long February–June precipitation from northern Pakistan has short- to medium-term (2–9.3 years) frequency cycles. The AMO, ENSO, PDO, and SOI are the major climatic modes/phases that influence the seasonal or annual climate variability or anomaly in the HKKH and adjacent regions^31,74–76^. The short periodicities of 2.2–8.3 years observed in our reconstruction fall in the band of ENSO cycles ^77,78^. These quasi-cyclic periodicities related to ENSO are some of the dominant forces to local dryness/wetness variation in the South Asian summer monsoon-dominated Himalaya and adjacent regions^6,7,27,28,30,34,79^. In four centuries long spring season streamflow reconstruction in Nepal using composite tree-ring residual chronologies they found predominantly high frequency signal related to ENSO. Another precipitation reconstruction from Myanmar also captured predominantly high frequency periodicity^80^. Ahmad et al.^57^ found periodicities of 2.1–2.4, 3.3, 6.0, 16.8, and 34.0–38.0 years in their 424-year March–August PDSI reconstruction in northern Pakistan. The decadal to multidecadal oscillations related to PDO and AMO in the precipitation have also been reported in the observed as well as reconstructed precipitation from the Himalaya region^24,26–28,30,81^. We found positive relationship between the reconstructed precipitation and ENSO indices (Nino3.4 and ONI) but relationship with other indices like AMO/NAO is weak in most of the months (Supplementary Table S3). Since we used residual chronologies for the reconstructions, we might lose some medium- and long-term periodicity cycles and mostly observed short periodicities in our reconstructions. When we tried reconstructions using standard chronologies, we also observed medium- to long-term (17.2, 24–29, 53–73 years) periodicities (Graph not shown). This could be the reason why we could not capture AMO-related periodicities that are observed in Ahmad et al.^21^ PDSI reconstructions from Pakistan and other precipitation from the Indian and Nepal Himalaya^27,28,82^. Panthi et al. ^82^ while doing spring season drought index reconstruction using standard chronologies captured AMO related signals. Our region is more influenced by western disturbance while the region of Panthi et al. ^82^ is primarily influenced by Indian summer monsoon. However, it is better to do further studies to disentangle the exact mechanism of how broader scale climate mode or phase affects the precipitation variability in the HKKH region in Pakistan and why different reconstructions have captured different spectral peaks in their reconstructions (artifact of methods used, or differential influence of circulation systems and climate models and phases). Morlet wavelet analysis revealed that the intensity of high frequencies is increasing in recent centuries and decades compared to the earlier period. Similarly, the frequency of extreme events is has also increased in our reconstructions during recent years, which is persistent in observed climatic extremes in the Himalayan region, including Pakistan^83^. Future studies can disentangle influence of western disturbance and nonwestern disturbance related precipitation during spring season and their behaviors^14^ and sensitivity of tree-ring to capture such influences. Study indicated that when the Himalayan jet latitude (HJL) is displaced poleward, most parts of central Asia experience anomalously hot and dry springs^84^. However, we found poor relationship between our reconstructed precipitation from northern Pakistan and reconstructed Himalayan jet latitudes during northward excursion years^84^. This demands more studies in the climate of the Himalayan region and their teleconnections with different climatic modes and their phases or circulation systems.

The findings of the present study have great significance and applications not only for Pakistan and but also for those regions influenced by similar climatic drivers especially for the water resource management in the context of climate change. Our study region in north-western Pakistan receives the majority of its annual precipitation during the winter and spring months/seasons in the form of snow and/or rain. We recorded several dry and wet periods during the past 635 years, along with increasing extremes in precipitation during recent years. In our reconstruction, 38 extremely wet and 38 extremely dry years were recorded. For example, one of the long-term droughts from 1998 to 2002 (we also recorded in our reconstruction) reduced agricultural production, with the largest reduction in wheat, barley, and sorghum (from 60 to 80%) ^55,57^. Northern Pakistan is considered to contain some of the world’s largest irrigation networks^85^. Such irrigation system could be frequently affected by increasing frequency of extreme (both dry and wet) climatic events. Extreme high precipitation events are often associated with floods, erosions and landslides in many areas^17^. Therefore, information of intensity and frequency of extreme precipitation events can be very useful in the preparedness for water induced disasters like floods (which are expected to increase in future climate change scenarios) in the Pakistan and adjacent regions. Similarly, several hydropower plants are situated in northern Pakistan. Therefore, the long-term regional precipitation reconstructions of the February-June season will help the planner tasked with water resource management and dealing with the hydropower management with proper planning of the water resource use. The information about the increasing precipitation extremes will also be useful for evidence-based decision-making and to prepare for the necessary adaptation measures to the on-going climatic extremes. Studies indicated towards decreasing influence of western disturbance in the winter and spring season over time^14^. But mechanism or driver of such increasing frequency of extreme events need to be studied and explored in the future studies.

## Conclusion

We reconstructed over half millennium long winter spring season (February-June) precipitation in the north-western Pakistan by using moisture sensitive regional composite tree-ring width chronology. Our over six-century February-June precipitation revealed dry and wet periods, along with increasing frequency of extremes in precipitation during recent centuries and decades. Our reconstruction effectively captures the precipitation variation in northern Pakistan and adjacent regions. We found possible teleconnections between the precipitation variability in northern Pakistan and broader-scale climate mode or phases like ENSO, SOI and AMO, however, exact mechanism of influence of different climatic phases and modes on the climate in the western Himalaya including Pakistan is the important topic for research in future. Similarly, being western disturbance main driver of the winter and spring season precipitation in western Himalaya, there is huge prospect to study how western disturbance is linked with this increasing intensity and frequency of extreme precipitation and the long-term atmospheric circulation variability over the Northern Pakistan and surrounding region using tree-ring data. Moisture sensitive tree-ring can be applied to explore the extent of historic drought events in the region as well as reconstruction of long-term ENSO events. The study has great significance for water resource management in Pakistan and adjacent regions in the context of rapid climate change and increasing climatic extremes in the HKKH region.

### Supplementary Information

Supplementary Information.

## Data Availability

Datasets are available from the senior author on reasonable request (N.K, first author).
