# Supplementary material for: Multi-century (635-year) spring season precipitation reconstruction from northern Pakistan revealed increasing extremes
Source: Sci Rep. 2024 Jan 2;14:92. doi: 10.1038/s41598-023-50819-5 (PMC10761852; doi:10.1038/s41598-023-50819-5)
Supplement: Supplementary file 1 — Supplementary Information. [file 41598_2023_50819_MOESM1_ESM.docx]

Table S1: Ecological characteristics and summary statistics of the TRW chronologies of *C*. *deodara* and *P*. *gerardiana* from the northern Pakistan. GLK= Gleichläufigkeit, AR1= autocorrelation of level 1; R-Bar= mean inter-series correlation; EPS=expressed population signal, MS= Mean sensitivity.

| Site Code | CD | CDAD | PG | Composite |
| --- | --- | --- | --- | --- |
| Species | *C. deodara* | *C. deodara* | *P. geradiana* | *C. deodara* and *P. gerardiana* |
| Latitude (N) | 35.886697 | 35.92397 | 35.9428833 |  |
| Longitude (E) | 71.7972 | 71.77523889 | 71.77620833 |  |
| No of cores | 168 | 41 | 41 | 241 |
| No of trees | 108 | 23 | 26 | 157 |
| First yr | 2018-1394 | 2017-1375 | 2017-1218 | 2018-1218 |
| Max. Age (yr) | 625 | 642 | 799 | 799 |
| Mean Age (yr) | 215.9 | 321.9 | 472.6 | 282.2 |
| Average radial growth (mm/yr) | 0.98 | 0.81 | 0.58 | 0.85 |
| R-bar | 0.57 | 0.60 | 0.59 | 0.57 |
| GLK | 0.68 | 0.70 | 0.69 | 0.67 |
| Gini coefficient | 0.20 | 0.20 | 0.19 | 0.20 |
| AR1 | 0.63 | 0.65 | 0.63 | 0.63 |
| EPS | 0.96 | 0.86 | 0.89 | 0.97 |
| MS | 0.24.6 | 0.237 | 0.253 | 0.25 |

Table S2: Result of Principal Component Analysis of site chronologies

| **Total Variance Explained** | | | |
| --- | --- | --- | --- |
| Component | Initial Eigenvalues | | |
|  | Eigenvalues | % of Variance | Cumulative % |
| 1 | 2.211 | 73.716 | 73.716 |
| 2 | .426 | 14.209 | 87.924 |
| 3 | .362 | 12.076 | 100.000 |
| Extraction Method: Principal Component Analysis. | | | |

Table S3: Relationship (Pearson’s correlation) between reconstructed FJ precipitation with different climate indices and modes for the common period of 1965 to 2017. Nino (3.4)=, SOI= Southern oscillation index, PDO= Pacific decadal oscillation, AMO= Atlantic multidecadal oscillation, NAO= north atlantic oscillation, ONI= ocenic nino index, MEI= Multivariate ENSO index.

|  | Previous year | | | | | | | | Current year | | | | | | | | | | | |
| --- | --- | --- | --- | --- | --- | --- | --- | --- | --- | --- | --- | --- | --- | --- | --- | --- | --- | --- | --- | --- |
| Indices | M | J | J | A | S | O | N | D | J | F | M | A | M | J | J | A | S | O | N | D |
| Nino(3.4) | 0.13 | 0.27 | 0.29 | 0.23 | 0.16 | 0.19 | 0.24 | 0.30 | 0.29 | 0.32 | 0.34 | 0.33 | 0.25 | 0.17 | 0.15 | 0.11 | 0.05 | 0.03 | 0.02 | 0.03 |
| SOI | -0.01 | -0.09 | -0.06 | -0.08 | -0.14 | -0.11 | -0.28 | -0.33 | -0.13 | -0.25 | -0.27 | -0.26 | -0.12 | 0.17 | 0.19 | 0.10 | 0.06 | 0.02 | -0.01 | 0.04 |
| PDO | -0.02 | -0.10 | 0.05 | 0.08 | 0.18 | 0.14 | 0.06 | -0.02 | 0.02 | 0.07 | 0.19 | 0.19 | 0.22 | 0.17 | 0.14 | 0.14 | 0.18 | 0.21 | 0.30 | 0.17 |
| NAO | 0.03 | -0.10 | -0.15 | 0.01 | 0.03 | -0.13 | -0.07 | 0.10 | 0.17 | -0.18 | -0.04 | 0.02 | 0.09 | 0.04 | 0.15 | 0.02 | -0.10 | -0.17 | 0.15 | -0.05 |
| ONI | 0.20 | 0.25 | 0.28 | 0.22 | 0.19 | 0.20 | 0.25 | 0.29 | 0.30 | 0.30 | 0.31 | 0.30 | 0.26 | 0.19 | 0.16 | 0.12 | 0.07 | 0.04 | 0.03 | 0.04 |
| MEI | 0.22 | 0.18 | 0.09 | 0.09 | 0.11 | 0.06 | 0.33 | -0.33 | 0.01 | 0.02 | -0.01 | 0.01 | -0.04 | -0.03 | -0.09 | -0.13 | -0.08 | -0.06 | -0.09 | 0.08 |
| AMO | -0.16 | -0.09 | -0.16 | -0.19 | -0.17 | -0.02 | -0.20 | -0.11 | -0.09 | -0.08 | -0.18 | -0.19 | -0.29 | -0.30 | -0.22 | -0.06 | -0.13 | -0.25 | -0.14 | -0.07 |


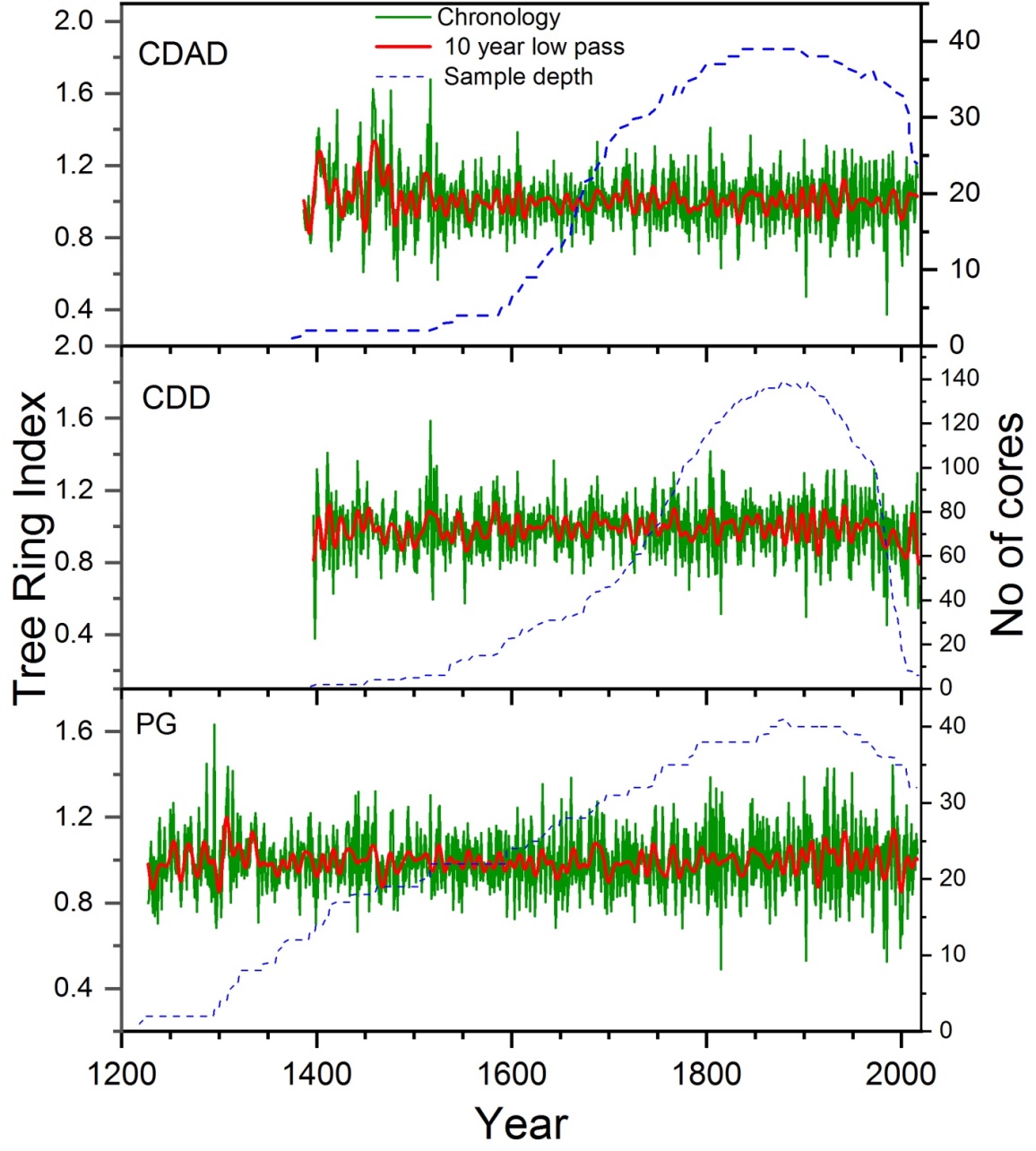


Figure S1: Tree-ring width residual site chronology (green) from Northern Pakistan along with their 10-year smoothing spline (thick red line), and sample depth or no of cores (blue dotted line) used in each chronology. The CDD and CDAD are the names of the individual site chronology of *C. deodara* while PG is the name of *P. gerardiana* chronology.


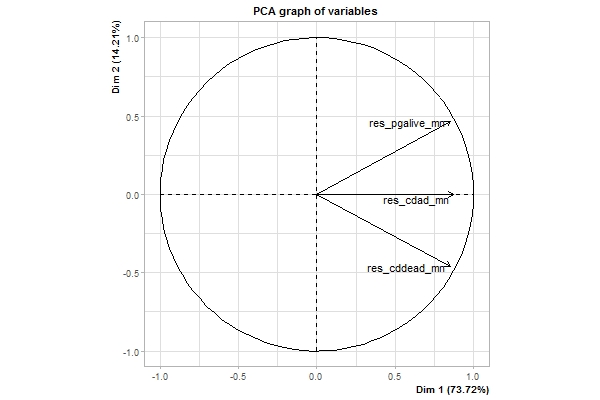


Figure S2: PCA of the residual tree-ring width site chronologies from northern Pakistan

**
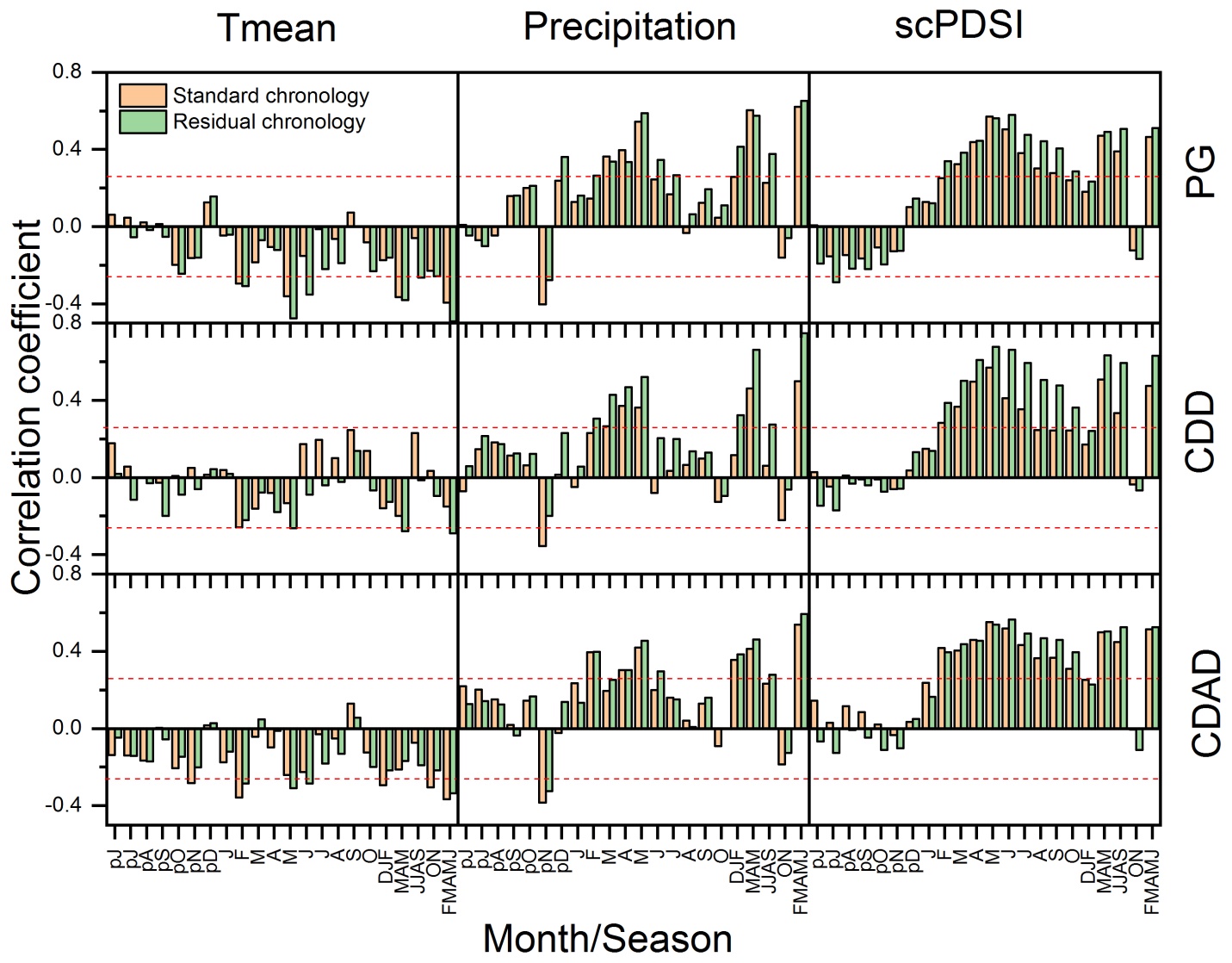
**

Figure S3: Relationship (Pearson's correlation coefficient) between the tree-ring site chronologies (standard and residual) and monthly and seasonal mean temperature, precipitation, and scPDSI data. The DJF, MAM, JJAS, ON represent winter, spring, summer, and autumn seasons. The FMAMJ (February-June) is the target season for reconstruction. The horizontal dashed line indicates 95% level of significance of the correlation coefficients. The PG, CDD, and CDAD are the name of individual site chronologies.

| Month and season | 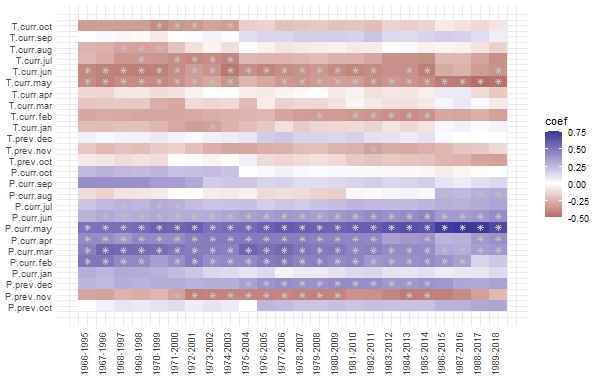 |
| --- | --- |
|  | Year |

Figure S4: Heat map showing moving correlation between the composite tree-ring chronology and Drosh station precipitation and mean temperature data from1965-2016. The * symbol in the map indicates significant correlation.


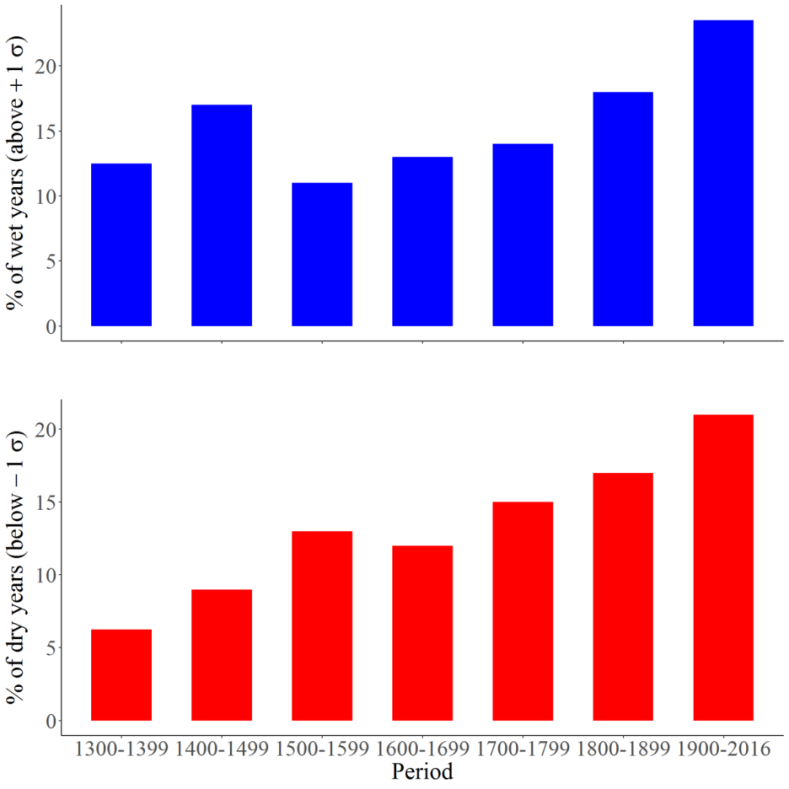


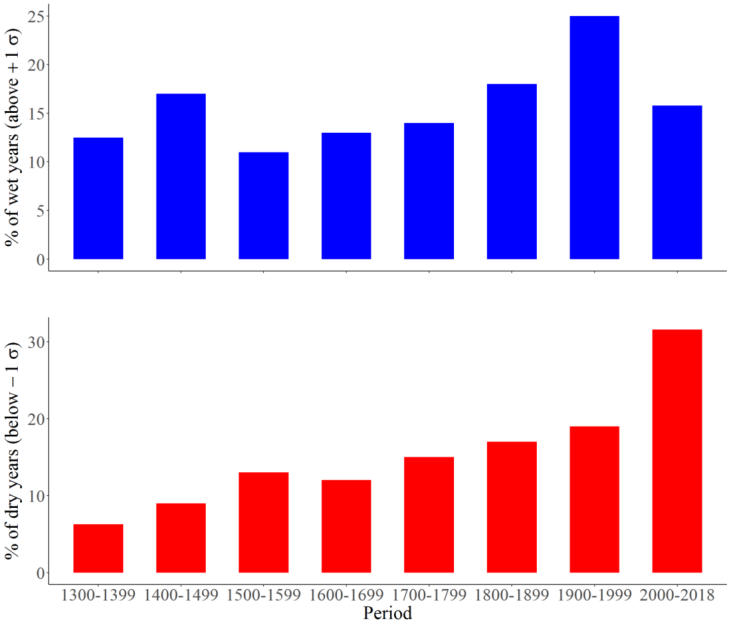


Fig.S5. Temporal change in the percentage of the extreme wet and dry events (mean± 1σ) in the reconstructed February-June precipitation from northern Pakistan.
